# Expression and Role of Oct3/4 in Injury-Repair Process of Rat Alveolar Epithelium after 5-Fu Treatment

**DOI:** 10.1155/2017/3856839

**Published:** 2017-08-29

**Authors:** Wen-ya Li, Xu-lv Ye, Xin-shan Jia, Lan-ling Jia

**Affiliations:** ^1^Department of Pathology, The First Affiliated Hospital of China Medical University, Shenyang, Liaoning Province, China; ^2^Department of Thoracic Surgery, The First Affiliated Hospital of China Medical University, Shenyang, Liaoning Province, China; ^3^Department of Pathology, College of Basic Medical Sciences, China Medical University, Shenyang, Liaoning Province, China

## Abstract

**Objective:**

We aimed to investigate how the embryonic stem cell-related gene Oct3/4 changes during the injury-repair process of distal pulmonary epithelium induced by 5-fluorouracil (5-Fu).

**Methods:**

We have developed the lung injury model induced by 5-Fu and observed the dynamic changes of Oct3/4 by indirect immunofluorescence, Western blot, and quantitative real-time PCR. Immunofluorescence double staining was used to compare the positions of Oct3/4(+) cells and other reported alveolar epithelial stem cells.

**Results:**

Oct3/4(+) cells were not found in normal rat lung epithelial cells. However, after treatment with 5-Fu, Oct3/4(+) cells appeared at 12 h, reached the peak at 24 h, then decreased at 48 h, and eventually disappeared at 72 h. Oct3/4 was localized in the nucleus. We found that the sites of Clara cell secretory protein and surfactant protein-C dual positive cells were apparently different from Oct3/4(+) cells.

**Conclusions:**

Our results revealed that, in rat alveolar epithelium, expression of Oct3/4 could be induced after treatment with 5-Fu, then decreased gradually, and was silenced following the alveolar epithelial differentiation. We hold that Oct3/4(+) cells are lung stem cells, which can provide new evidence for identification and isolation of lung epithelial stem cells.

## 1. Introduction

Lung stem cells (LSCs) refer to the cells with the capacity to self-renew constantly and to differentiate into a variety of lung tissues. The complicated components of pulmonary epithelium and mesenchymal cells, which amount to more than 40 types [1, 2] in addition to low self-renewal and limited regenerative ability of pulmonary epithelium, lead to the slower progress on LSCs compared to stem cells of other organs. It has been reported that LSCs can accelerate the division after severe lung injury, give birth to secondary stem cells and progenitor cells of various tissues and cells, which finally differentiate into functional cells, take place of the injured cells, and repair and heal the wound [3]. Over the past few years, many cells have been found to exhibit the characteristics of LSCs during development and play a role in lung injury repair. In 1969, Kaplan et al. reported that alveolar epithelial cell (AEC) II proliferated and differentiated into cells with features of AEC I 4 days after oxygen toxicity in monkeys, covering exposed collagen, replacing damaged AEC I [4]. Based on the findings, they considered AEC II as stem cells of alveolar epithelium. Cultured in vitro, AEC II lost their surface markers such as surfactant protein-C (SPC), expressed with AEC I specific markers, and transformed from cubic cells into flattened cells [5]. Another researcher found a subpopulation of variant Clara cell secretory protein (CCSP) cells, to represent LSCs. Due to lack of cytochrome P450 isoenzyme in cytoplasm, these cells did not take part in metabolism of naphthalene (one of the toxic components in cigarette smoke), thereby having resistance to naphthalene injury. Most importantly, these cells could proliferate and differentiate into other types of distal airway cells [6]. Kim and colleagues found that a subtype of AEC II, at the junction between the conducting and respiratory epithelium (the bronchioalveolar duct junction, BADJ), proliferated rapidly and differentiated into AEC I when lung got injured. These cells, coexpressed with SP-C, CCSP, Sca-1, and CD34, without expression of CD31 or CD45, were resistant to naphthalene and bleomycin [7].

Oct3/4 is a synonym for Pou5f1 which encodes Pou5f1 protein, a member of the POU family of transcription factors [8, 9]. Oct3/4 is regarded as a well-known marker of totipotency, due to its vital role in maintenance of self-renewal and undifferentiated state in stem cells [10–14]. It is reported to be expressed in many kinds of totipotent cells including oocytes, archaeocytes, preimplantation embryos, primitive ectoderm, inner cell mass, and embryonic stem cells [15–18] and rarely expressed in differentiated cells [13, 14, 19].

Our team first established the rat repair model of tracheal injury caused by 5-Fu [20–23] and found that, after being treated with 5-Fu, proliferating tracheal epithelium showed degeneration and necrosis, and the residual G0 cells in the basement membrane expressed embryonic stem cell-related genes such as Oct3/4, Sox2, and Nanog. However, after being differentiated into basal cells, ciliated cells, and mucous cells, expression of Oct3/4, Sox2, and Nanog disappeared. No study has been reported on the changes of stem cell-related gene Oct3/4 in the injury and repair process of distal pulmonary epithelium induced by 5-Fu. Using 5-Fu injury model, this study was focused on the dynamic changes of Oct3/4 in the repopulation process, which may provide new evidence for the identification and isolation of lung epithelial stem cells.

## 2. Materials and Methods

### 2.1. Rats and Tissues

Male and female Wistar rats (~200 g) were used in accordance with the guidelines of the Animal Care Committee of the China Medical University. After ether anesthesia and tracheal incubation, 5-Fu was administered intratracheally at the dose of 10 mg/kg, and the same amount of PBS was used as control. After 5-Fu treatment, rats were killed at 12 h, 24 h, 48 h, and 72 h, respectively, and certain parts of lung were collected under sterile conditions. Some were used for HE staining or immunofluorescence staining and others for Western blot analysis, which were stored at −80°C until further use.

### 2.2. Indirect Immunofluorescence

Indirect immunofluorescence staining was performed using Oct3/4 antibodies, on serial sections (4 um thickness) of lung tissue, using an experimental protocol as described previously [22]. Briefly, rabbit anti-Oct3/4 (dilution 1 : 100; Santa Cruz Biotechnology, Santa Cruz, CA,USA) were used as primary antibodies. Rhodamine isothiocyanate (TRITC)-conjugated goat anti-rabbit IgG (dilution 1 : 100) were used as secondary antibodies, which were diluted with 1% bovine serum albumin- (BSA-) PBS. After treatment with the secondary antibody, specimens were incubated with 0.5% ug/mL of 4,6-diamidino-2-phenylindole (DAPI; Sigma) for nuclear counterstaining. Specimens were examined using an epi-illumination fluorescence microscope BX50 (Olympus, Tokyo, Japan). For serum controls, 1% BSA-PBS instead of the primary antibody was used as a negative control.

### 2.3. Immunofluorescence Double Staining

We took the lung tissues treated with 5-Fu after 24 h for immunofluorescence double staining. The paraffin-embedded tissues were cut into 4 *μ*m thick slides, dewaxed in xylene, and dehydrated in graded alcohols. The antigen was retrieved by heating for 90 seconds in 0.01 mol/L citrate buffer (PH 6.0), followed by blocking administrated using nonspecific normal serum. The specimens were incubated separately with primary antibodies, anti-Nanog antibody (species: mouse, 1 : 100) (Santa Cruz Biotechnology, Inc., Santa Cruz, CA, USA), anti-SP-C antibody (species: rabbit, 1 : 100) (Santa Cruz Biotechnology, Inc., Santa Cruz, CA, USA), and anti-CCSP antibody (species: mouse, 1 : 100) (Santa Cruz Biotechnology, Inc., Santa Cruz, CA, USA) overnight at 4°C. The two secondary antibodies were fluorescein isothiocyanate- (FITC-) conjugated anti-rabbit IgG and tetramethylrhodamine- (TRITC-) conjugated anti-mouse IgG in light-tight condition, and the nuclei were stained by DAPI (Sigma-Aldrich). For negative control, 1% BSA in PBS without primary antibody was used. The specimens were examined using a BX51 inverted epifluorescence microscope (Olympus).

### 2.4. Western Blot Analysis

The cell lysate was prepared by NP40 lysis buffer containing 20 mM Tris–HCl (pH 8.0), 137 mM NaCl, 1% NP40, 10% glycerol, and 4% complete protease inhibitor cocktail mix (Roche, Mannheim, Germany). 60 *μ*g protein was used for sodium dodecyl sulphate–polyacrylamide gel electrophoresis and transferred blotting to polyvinylidene fluoride (PVDF) (Immobilon; Millipore Corp, Billerica, MA, USA). Membranes were blocked using TBST solution containing 4% skim milk for 1 h with gentle shaking and were then washed three times for 15 min with TBST solution. Membranes were incubated overnight at 4°C with rabbit anti-Oct3/4 (dilution 1 : 500), followed by shaking in TBST. After washing, membranes were incubated with secondary antibodies for 2 h at room temperature. While washing repeatedly as described earlier, membranes were incubated with ECL for 1 min at room temperature. We detected the protein using the BioImaging Systems (UVP Inc., Upland, CA, USA), when bands reached the desired darkness. Relative amount of protein was quantified with the reference of *β*-actin (dilution 1 : 1000, Santa Cruz Biotechnology, Santa Cruz, CA,USA).

### 2.5. Quantitative Real-Time PCR

Total RNA was extracted using TRIzol reagent (Invitrogen, Carlsbad, CA, USA). Reverse transcription was performed with cDNA RT kit (Applied Biosystems, Foster city, CA, USA), and quantitative real-time PCR was performed using real-time PCR system (Applied Biosystems). *β*-Actin was used as internal control. Relative mRNA expression of Oct3/4 was calculated using comparative Ct-method when normalized to *β*-actin expression levels. The primers sequences were as follows: Oct3/4 forward, 5′-CGCAAGCCCTCATTTCAC-3′, Oct3/4 reverse, 5′-CATCACCTCCACCACCTG-3′; *β*-actin forward, 5′-ATAGCACAG CCTGGATAGCAACGTAC-3′, *β*-actin reverse, 5′-CACCTTCTACAA TGAGCTGCGTGTG-3′ [24].

### 2.6. Statistical Analysis

SPSS 16.0 (SPSS Inc., Chicago, IL, USA) was used for statistical analysis. All values were expressed as mean ± SD. Statistical analyses were performed using one-way ANOVA and a *p* value of <0.05 was considered as statistically significant. 

## 3. Results

### 3.1. Morphological Changes in Rats Pulmonary Alveolar Epithelium after 5-Fu Treatment

**Figure 1:**
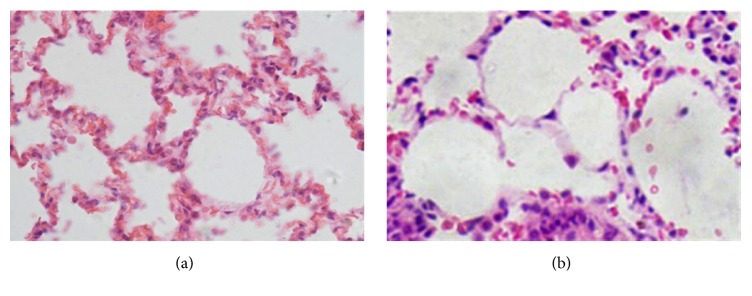
Morphological changes in rat alveolar epithelium before and after injury induced by 5-Fu as observed by HE staining. (a) Normal rat alveolar tissue is shown. (b) After 5-Fu treatment, only a few alveolar epithelial cells remained (original magnification ×400).

The normal alveolar epithelium in rats, mainly consisting of AEC I and AEC II, was characterized by structural integrity, uniform shapes, and clear alveolar contour. However, after 5-Fu treatment, alveolar structure damaged and manifested with degeneration, necrosis, and detachment of alveolar cells, leaving only few residing, and infiltration of inflammatory cells into mesenchyma (Figure 1).

### 3.2. Expression of Oct3/4 in Alveolar Epithelium before/after Treatment with 5-Fu by Immunofluorescence Staining

**Figure 2:**
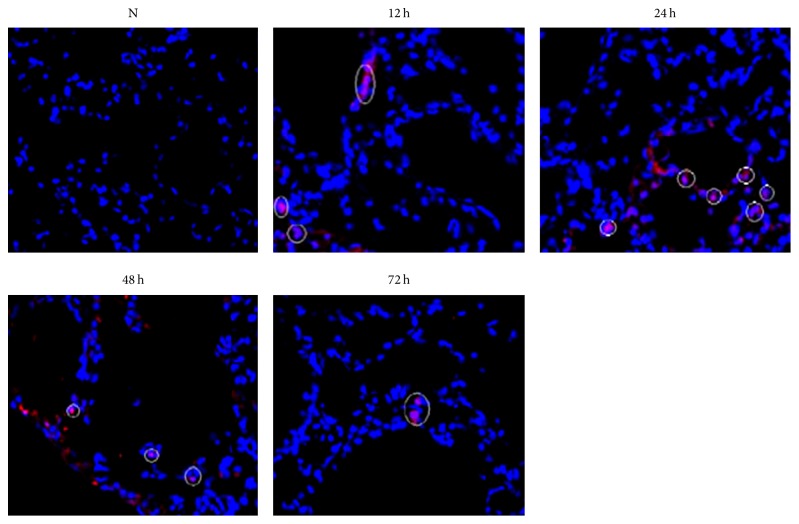
Expression of Oct3/4 in alveolar epithelium by immunofluorescence staining. Normal rat alveolar epithelium, no Oct3/4 (+) cells (N). Few Oct3/4 (+) cells began to appear (12 h). Oct3/4 (+) cells reached the peak (24 h). Oct3/4 (+) cells decreased gradually (48 h). Oct3/4 (+) cells were close to the normal level (72 h). The circled cells: Oct3/4 (+) cells (original magnification ×400).

In normal lung epithelial cells, there were no Oct3/4 (+) cells. After 5-Fu treatment, Oct3/4 (+) cells began to emerge after 12 h, reached the maximum at 24 h, decreased after 48 h, and were almost equivalent to normal level after 72 h (Figure 2, Table 1). Oct3/4 (+) was localized in the nucleus.

### 3.3. Expression of Stem Cell Marker Oct3/4 in Alveolar Epithelium as Detected by Western Blot Analysis and Quantitative Real-Time PCR

**Figure 3:**
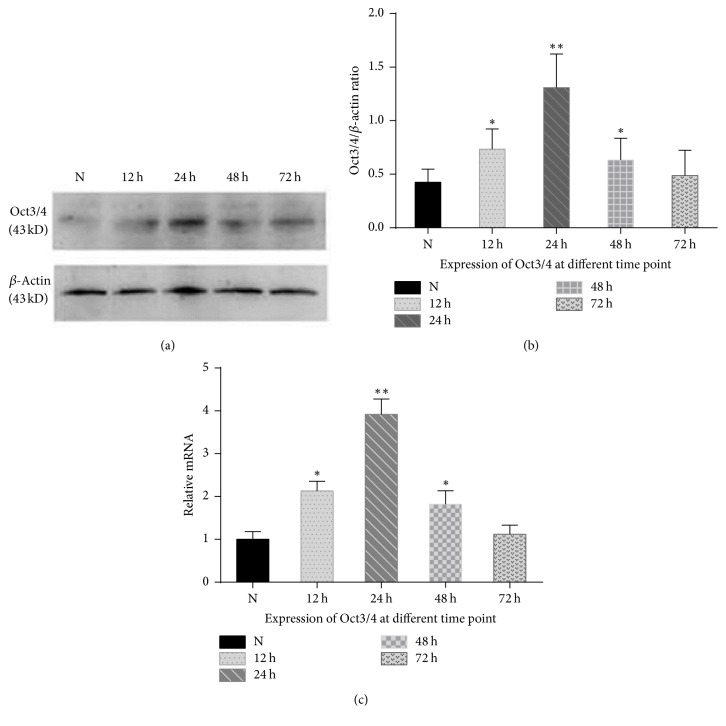
Expression levels of Oct3/4 protein in rat alveolar epithelium during recovery from 5-Fu induced injury. ((a) and (b)) Western blot analysis of Oct3/4 protein in normal lung and 5-Fu-treated lung. (c) Relative mRNA expression of Oct3/4 at different time point by quantitative real-time PCR. Data are presented as the mean ± SD of three independent experiments. ^*∗*^*p* < 0.05 and ^*∗∗*^*p* < 0.01 relative to levels in untreated control mice.

To confirm changes of Oct3/4, the protein level of Oct3/4 was examined using Western blot analysis and quantitative real-time PCR (Figure 3). Oct3/4 was not detectable in normal rat lung epithelium. After treatment with 5-Fu, its expression level increased and reached the maximal level after 24 h and decreased gradually and after 72 h returned to levels similar to untreated situation. These results coincided with the observations obtained by immunofluorescence staining.

### 3.4. Comparison of Locations of Oct3/4 Positive Cells with SP-C and CCSP Cells by Immunofluorescence Double Staining

**Figure 4:**
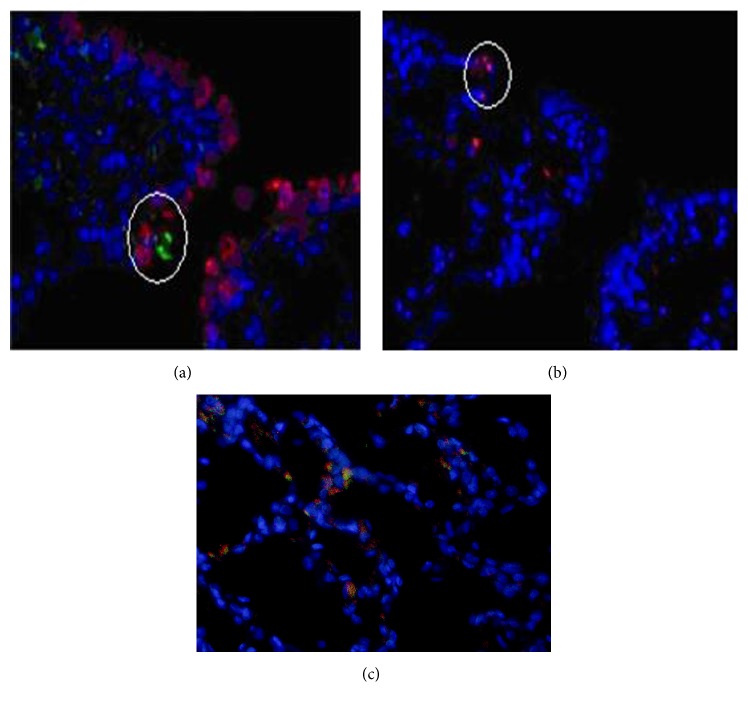
Location of Oct3/4, SP-C, and CCSP positive cells in rat alveolar epithelium. (a) Immunofluorescence double staining was used, where green fluorescence stands for SP-C positive cells and red fluorescence for CCSP potive cells. (b) Indirect immunofluorescence performed on serial slice showed Oct3/4 positive cells with red fluorescence. (c) Nanog was copositively expressed in Oct3/4 positive cells, where green fluorescence stands for Nanog positive cells and red fluorescence for Oct3/4 potive cells (original magnification ×400).

To further clarify whether Oct3/4 positive cells are the same as SP-C and CCSP positive cells, we used immunofluorescence double staining, by which we found that the position of Oct3/4(+) cells was different from that of SP-C and CCSP positive cells. Besides, another pluripotency marker, Nanog, was also detected in Oct3/4(+) cells, which were found copositively expressed (Figure 4).

## 4. Discussion

It is believed that adult stem cells mainly exist in a state of nonproliferation, reversible cell cycle-arrest, known as quiescence (G0 phase) [25]. Being quiescent, it may help to preserve a small and steady amount of stem cells for regeneration against injury, working as a protective mechanism from the point of evolution [26, 27]. The chemotherapeutic agent, 5-Fu, is a pyrimidine analogue that inhibits thymidylate synthase, a necessary synthesis enzyme for DNA synthesis. 5-Fu specifically targets proliferating cells, and G0 phase cells can be spared due to 5-Fu induced cell death [28, 29]. Thus, theoretically and practically, it is reasonable to select and enrich stem cells by 5-Fu treatment [23, 28, 30].

Oct3/4 was absent in rat normal alveolar epithelium. However, after treatment with 5-Fu, the mature cells in proliferation underwent degeneration and necrosis; only 5-Fu resistant cells in G0 stage survived on the alveolar wall, which were partially expressed with Oct3/4. When large area of alveoli was injured, Oct3/4 (+) cells began to increase and proliferate in order to repopulate the damaged epithelium on the basement membrane. After being differentiated into Clara cells and AEC I and AEC II cells, Oct3/4 was gradually silenced, together with the observation that Nanog was copositively expressed in Oct3/4(+) cells, which may illustrate that Oct3/4(+) cells were undifferentiated or with multidifferentiation ability. Once the alveolar epithelium recovered, the expression of Oct3/4 almost completely disappeared. Oct3/4 was originally found to be expressed in embryonic stem cells, and its inactivation leads to loss of pluripotency and apoptosis [13]. Tai et al. have shown that Oct3/4 expression was found in adult human kidney, mesenchymal, breast epithelial, liver, and gastric stem cell lines, and when induced to differentiate, Oct3/4 obviously decreased and diminished [31]. So Oct3/4 can be used as a specific marker for LSCs.

In 2005, Kim et al. reported that, in their mice tumor model induced by oncogenic K-ras, there was a population of cells at BADJ, which coexpressed CCSP and SPC, termed double-positive cells (DPCs). DPCs, marked by CD45^−^CD31^−^CD34^+^Sca-1^+^, were considered as bronchioalveolar stem cells in mice [7]. However, in the literature, such cells were only observed in specific conditions, such as lung cancer tissues and in vitro experiments [32]. So far, DPCs have not been detected in human normal lung tissues. In this study, the location of Oct3/4 (+) cells was quite different from the DPCs'. Clara cells and AEC II cells were also recognized as lung stem cells. But both of these cells widely exist in lungs, accounting for a large part of distal lung tissue; how can it be possible that there are so many stem cells in pulmonary epithelium? In addition, Clara cells have secretory granules, secreting protease and many enzymes, and AEC II cells can release surface active substances. Their specific cellular function means they are terminally differentiated, which is contrary to the definition of stem cells being undifferentiated and multipotent. Since cells with specific secretory function cannot be viewed as stem cells, then it is hard to accept DPCs, expressing both CCSP and SPC, as LSCs.

We acknowledge that the ultimate test for stem cell activity is a single-cell clonogenic transplantation assay, as has been done in the hematopoietic system [33]. However, such assays are particularly challenging when applied to stem cells of epithelial tissues. Epithelial cells are closely associated with endothelial cells, stromal fibroblasts, inflammatory cells, and accompanying extracellular matrix and cell-cell interactions. So further research is needed.

In brief, our data indicated that Oct3/4 was absent in normal rat alveolar epithelium. However, when it is treated with 5-Fu, Oct3/4 expression increased in alveolar epithelium. After being differentiated into various types of alveolar cells, Oct3/4 decreased gradually and was silenced finally. Clara cells and AEC II cells do not match the definition of stem cells, due to not only their large number but also their specific secretory function. We believe that Oct3/4 (+) cells are alveolar epithelial stem cells. This study provides new insight and experiment basis for the isolation, purification, and committed differentiation of LSCs.

## Figures and Tables

**Table 1:** The number of Oct3/4 positive cells and total number of cells before/after treatment with 5-Fu (per five 400x views).

	N	12 h	24 h	48 h	72 h
Oct3/4 (+) cells	0	34	40	38	13
Total number	321	180	124	226	311
Ratio (%)	0	18.9	32.3	16.8	4.2

Ratio = Oct3/4 (+) cells/total cells.
